# Vertebral artery transection with pseudoaneurysm and arteriovenous fistula requiring antegrade and retrograde embolization

**DOI:** 10.1016/j.jvscit.2022.01.009

**Published:** 2022-03-03

**Authors:** Maham Karatela, E. Hope Weissler, Mitchell W. Cox, Zachary F. Williams

**Affiliations:** Division of Vascular and Endovascular Surgery, Duke University School of Medicine, Durham, NC

**Keywords:** Arteriovenous fistula, Contralateral, Endovascular, Traumatic injury, Vertebral artery

## Abstract

Traumatic vertebral artery injury is a rare, life-threatening injury that has been increasingly managed with endovascular intervention. However, an antegrade endovascular approach alone can fail to occlude traumatic pseudoaneurysms (PSAs) and arteriovenous fistulas (AVFs), requiring high-risk surgical reoperation. We have presented the case of a 27-year-old man with traumatic right vertebral artery PSA and AVF. Despite successful ipsilateral coil embolization, the PSA and AVF persisted via retrograde filling from the contralateral vertebral artery. Distal coil embolization was achieved through the contralateral vertebral artery in a novel “up and over” approach through the basilar artery. The findings from our case report have broadened the endovascular options for complicated traumatic injuries.

Traumatic vertebral artery injury (VAI), from a hematoma with luminal stenosis to pseudoaneurysms (PSAs), arteriovenous fistulas (AVFs), and complete occlusion, has been reported in <1% of blunt and penetrating trauma admissions but is highly morbid, with a stroke risk of 6% to 38%.^1^, ^2^, ^3^, ^4^ The estimated mortality directly associated with traumatic VAI has been reported at 5% to 7% and, when untreated, can reach 70% for severe injuries.^1^^,^^3^^,^^5^

The treatment options include observation and antithrombotic therapy for minor intimal injuries; open surgery for accessible high-flow AVFs, PSAs, and vessel transections; and endovascular intervention for surgically inaccessible PSAs.^1^ Although the V1 segment can be more easily exposed than the V2 to V4 segments, 50% of these injuries occur in segments V2 to V4.^5^ Endovascular approaches have had better success and decreased morbidity compared with conservative management or open surgery.^1^^,^^5^^,^^6^ Usually, the lesion will be approached ipsilaterally; however, this approach can fail when encountering difficulty crossing lesions or when retrograde collateral flow has continued to perfuse an injured segment.

Failure of endovascular treatment has been most commonly followed by surgical reoperation. We have presented a case in which failure of antegrade embolization of a traumatic vertebral artery transection with an associated PSA and AVF was managed through retrograde embolization from the contralateral vertebral artery. The patient provided written informed consent to the report of his case details and imaging studies.

## Case report

**Fig 1:**
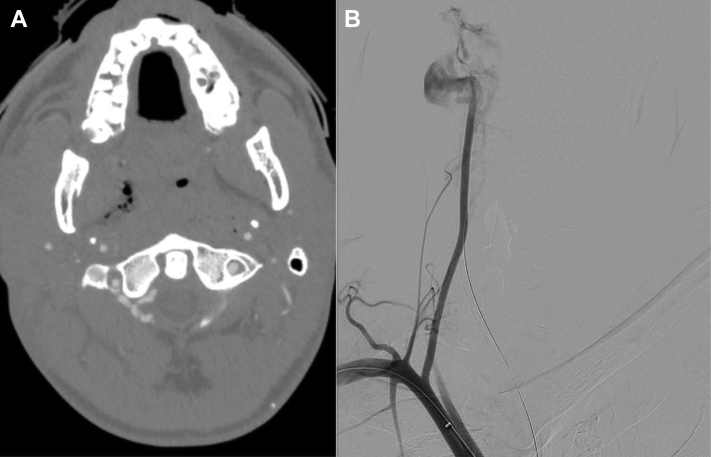
Computed tomography angiogram **(A)** and arteriogram **(B)** revealing active extravasation from the right vertebral artery.

A healthy 27-year-old man had presented to the emergency department after a stab injury to zone 3 of the right neck with a wound at the posterior right cheek. He presented in significant respiratory distress with a firm, expanding hematoma and a pulsatile hemorrhage from his right lateral neck. He was intubated, and a large figure-of-eight suture was placed, controlling the site of the hemorrhage. Once the airway was secured and external bleeding had been controlled, computed tomography angiography of the head and neck was obtained for operative planning during the setup of the hybrid operating room. The computed tomography angiogram demonstrated a large, extracranial, right vertebral artery PSA with active extravasation, a dissection flap, and a traumatic vertebral AVF in the V2 segment at the level of C3 (Fig 1, *A*). Antegrade, endovascular embolization for PSA and AVF treatment was favored over open surgery, given the difficulty of exposing V2 and V3 during active hemorrhage.

**Fig 2:**
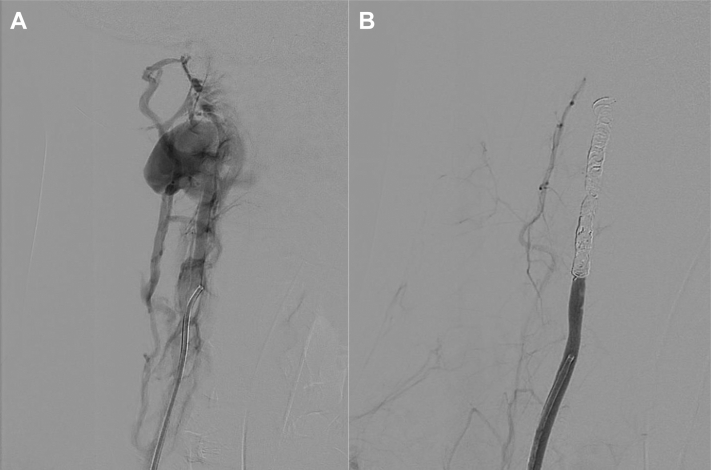
Before **(A)** and after **(B)** antegrade embolization of the transected right vertebral artery.

In the operating room, the right common femoral artery was accessed. A 5F sheath was placed in the innominate artery, and a Berenstein catheter was placed in the right vertebral artery. Cerebral arteriography demonstrated extravasation from the right vertebral artery, with no ipsilateral carotid artery injury (Fig 1, *B*). Despite multiple attempts, the area of injury could not be crossed. The right vertebral artery was coiled proximally to stop the acute bleeding. The completion angiogram demonstrated successful coil embolization, with no ongoing extravasation (Fig 2, *A and B*).

The patient was transferred to the neurologic intensive care unit for postprocedure monitoring. Later the same day, he developed partial right abducens nerve palsy and absent right corneal light reflexes suspicious for brainstem ischemia. Overnight, a brain magnetic resonance imaging study was negative for acute ischemic stroke. However, repeat computed tomography angiography demonstrated early venous filling of the right spinal venous plexus, raising concern for a persistent AVF causing a steal phenomenon.

**Fig 3:**
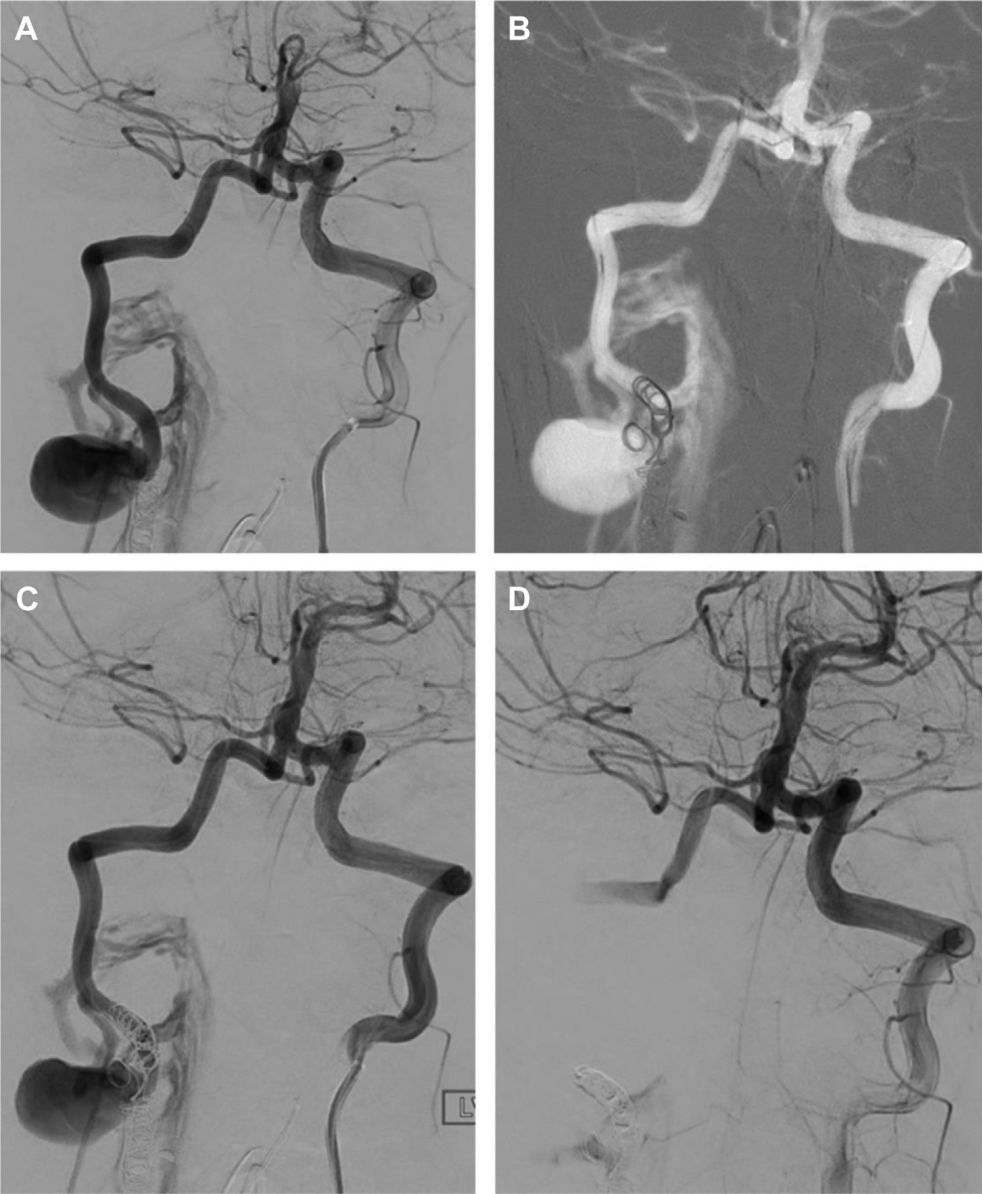
Intraoperative angiograms. **A,** Posterior cerebral arteriogram revealing large right pseudoaneurysm (PSA) and arteriovenous fistula (AVF) with no antegrade into the basilar artery. **B,** Coil placement. **C and D,** Additional coil placement with eventual stasis in vertebral artery and antegrade flow into basilar artery.

In the morning, the patient underwent repeat angiography to further delineate the persistent AVF. After accessing the right femoral artery, selective angiogram of the right vertebral artery demonstrated complete occlusion of antegrade blood flow beyond the coil embolization. Selective angiogram of the left vertebral artery demonstrated a large PSA arising from the V2 segment of the right vertebral artery, with an AVF draining into the epidural venous plexus (Fig 3, *A*). After a multidisciplinary discussion with neurosurgery, an additional endovascular attempt with angiographic guidance was deemed reasonable before open intervention, which would require suboccipital craniectomy.

Retrograde embolization was performed by accessing the left vertebral artery and traversing the basilar artery. A 5F catheter was advanced into the distal left V2 segment. Next, a Headway 17 microcatheter (Terumo Corp, Tokyo, Japan) and Synchro-2 microwire (Stryker Neurovascular, Fremont, CA) combination was advanced up the basilar artery and down the right vertebral artery adjacent to the PSA and AVF. Five embolic coils were deployed. Final anteroposterior and lateral angiograms demonstrated complete occlusion of the PSA and AVF with stasis within the right vertebral artery (Fig 3, *B-D*). The patient’s symptoms of brainstem ischemia had resolved completely. He was extubated the next day, and his remaining hospital course was unremarkable.

## Discussion

VAI from penetrating trauma is rare and life-threatening.^1^ The proximal vertebral artery (V1) is relatively surgically accessible.^7^ As the V1 enters the C6 transverse foramen and becomes V2,^8^ exposure requires segmental osteotomies of the C1 to C6 transverse processes,^7^ which can be challenging in the presence of active hemorrhage. Distal vascular control of the V3 segment, spanning from the C1 transverse foramen to the skull base, can require suboccipital craniectomy.^7^

Given the difficulty of surgical exposure, VAIs have been increasingly managed with endovascular interventions, which have decreased morbidity and mortality.^5^^,^^6^^,^^9^ Coil embolization is preferred for V3 transection and is safe if the contralateral vertebral artery is patent and has anatomic potential for retrograde filling of the distal intracranial vertebral artery branches.^10^^,^^11^ If the contralateral vertebral artery is occluded, covered stent placement or bare metal stenting with embolization through stent fenestrations are options, although no absolute contraindication exists to coil embolization.^12^^,^^13^ Herrera et al^14^ described 18 penetrating VAIs (16 AVFs and 2 PSAs) that were managed with balloon occlusion, coil embolization, and/or stent placement, with 100% success and no complications. In their study, 88.8% of patients had had vertebral AVFs, of which 43.7% had had coexisting PSAs. The high incidence of AVFs in patients with penetrating VAIs likely results from the paired veins with extensive collaterals adjacent to the vertebral artery.^6^

Typically, proximal ligation or embolization can resolve life-threatening hemorrhage. Obtaining distal control, surgically and endovascularly, can be difficult for transection or high-volume AVFs. Endovascular treatment failure will generally result from failure of proximal embolization, requiring subsequent open intervention. In a study of 22 patients with a VAI, 3 patients with residual PSAs and AVFs after proximal embolization had required suboccipital craniectomy for distal ligation.^9^ In 101 patients with extracranial VAIs (92 from penetrating wounds), 5 of the 33 patients who had undergone coil embolization had required subsequent surgery. Two patients had had high-flow AVFs requiring distal ligation, and three had undergone unsuccessful PSA embolization. Another two patients had required open surgery after becoming unstable during angiography, one of whom had sustained an iatrogenic vertebral artery laceration and died postoperatively.^5^

Up and over retrograde access via the contralateral vertebral artery offers another method for addressing incomplete VAI treatment that avoids challenging and more invasive surgical reoperation. Retrograde vertebral access has been performed for other indications, most often for vertebral or posterior inferior cerebellar artery aneurysms that cross the midline or cannot be accessed ipsilaterally for reasons that include subclavian stenosis.^15^, ^16^, ^17^, ^18^, ^19^ However, this technique has only rarely been applied in the setting of penetrating trauma.^20^^,^^21^ Retrograde vertebral access can be especially useful for polytrauma patients who are too unstable for time-consuming, complex, open surgery.

## Conclusions

VAI is rare, although the treatment options have been continually expanding. The findings from the present case report has demonstrated successful treatment of a symptomatic traumatic vertebral artery PSA and AVF with antegrade and retrograde embolization. Contralateral, retrograde access to the distal vertebral artery broadens the endovascular options for complicated injuries and might offer decreased morbidity compared with open surgery.
